# *In Vivo* Quantification of Placental Insufficiency by BOLD MRI: A Human Study

**DOI:** 10.1038/s41598-017-03450-0

**Published:** 2017-06-16

**Authors:** Jie Luo, Esra Abaci Turk, Carolina Bibbo, Borjan Gagoski, Drucilla J. Roberts, Mark Vangel, Clare M. Tempany-Afdhal, Carol Barnewolt, Judy Estroff, Arvind Palanisamy, William H. Barth, Chloe Zera, Norberto Malpica, Polina Golland, Elfar Adalsteinsson, Julian N. Robinson, Patricia Ellen Grant

**Affiliations:** 1 Fetal-Neonatal Neuroimaging and Developmental Science Center, Boston Children’s Hospital, Boston, 02115 USA; 2 Madrid-MIT M+Vision Consortium, RLE, Massachusetts Institute of Technology, Cambridge, 02139 USA; 3 Maternal Fetal Medicine, Brigham and Women’s Hospital, Boston, 02115 USA; 4 Pathology, Massachusetts General Hospital, Boston, 02114 USA; 5 Radiology, Massachusetts General Hospital, Boston, 02114 USA; 6 Radiology, Brigham and Women’s Hospital, Boston, 02115 USA; 7 Radiology, Boston Children’s Hospital, Boston, 02115 USA; 8 Anaesthesia, Brigham and Women’s Hospital, Boston, 02115 USA; 9 Obstetrics and Gynecology, Massachusetts General Hospital, Boston, 02114 USA; 10 Medical Image Analysis and Biometry Laboratory, Universidad Rey Juan Carlos, Madrid, 28933 Spain; 11 Electrical Engineering and Computer Science, Massachusetts Institute of Technology, Cambridge, 02139 USA; 12 Computer Science and Artificial Intelligence Laboratory, Massachusetts Institute of Technology, Cambridge, 02139 USA; 13 Institute for Medical Engineering and Science, Massachusetts Institute of Technology, Cambridge, 02139 USA

## Abstract

Fetal health is critically dependent on placental function, especially placental transport of oxygen from mother to fetus. When fetal growth is compromised, placental insufficiency must be distinguished from modest genetic growth potential. If placental insufficiency is present, the physician must trade off the risk of prolonged fetal exposure to placental insufficiency against the risks of preterm delivery. Current ultrasound methods to evaluate the placenta are indirect and insensitive. We propose to use Blood-Oxygenation-Level-Dependent (BOLD) MRI with maternal hyperoxia to quantitatively assess mismatch in placental function in seven monozygotic twin pairs naturally matched for genetic growth potential. In-utero BOLD MRI time series were acquired at 29 to 34 weeks gestational age. Maps of oxygen Time-To-Plateau (TTP) were obtained in the placentas by voxel-wise fitting of the time series. Fetal brain and liver volumes were measured based on structural MR images. After delivery, birth weights were obtained and placental pathological evaluations were performed. Mean placental TTP negatively correlated with fetal liver and brain volumes at the time of MRI as well as with birth weights. Mean placental TTP positively correlated with placental pathology. This study demonstrates the potential of BOLD MRI with maternal hyperoxia to quantify regional placental function *in vivo*.

## Introduction

Monitoring placental function *in vivo* and in real time is crucial for optimizing fetal and maternal outcomes as placental dysfunction has been linked to numerous fetal and maternal disorders from intrauterine demise to maternal cardiovascular disease^1^. Current clinical management of suspected placental dysfunction often relies on ultrasound assessment of fetal size and amniotic fluid volume as well as Doppler assessments of blood flow in the umbilical arteries and other fetal vessels to indirectly assess placental function. None of these measurements offers direct information about gas or nutrient exchange in the placenta^2^. The ability of these tests to distinguish the fetus with low genetic growth potential from the one compromised by placental dysfunction is limited. Since early delivery is often recommended for placental dysfunction, determining who should be delivered and when remains a challenge, as preterm birth is also associated with unfavorable outcomes^3, 4^. It is plausible that direct evaluation of regional placental function *in vivo* could improve diagnostic accuracy and risk stratification when there is a suspicion of placental dysfunction. In monochorionic (therefore monozygotic) twins, the genetic growth potential, gestational age, as well as maternal environmental factors are naturally controlled within each twin pair. This provides an ideal opportunity to investigate the potential of new placental biomarkers, since discordance in fetal growth can be more clearly attributed to twin differences in placental function.

**Figure 1:**
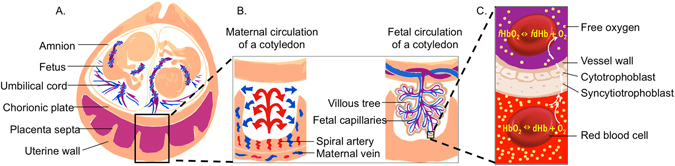
Schematic diagrams depicting (**A**) the main components of a monochorionic diamniotic twin gestation. The magnified views in (**B**) represent the maternal placental circulation (left) and the fetal placental circulation (right) with an inset cartoon (**C**) representing oxygen transport from mother to the fetuses. Oxygen exchange between maternal hemoglobin and fetal hemoglobin by free diffusion across the interhemal membrane, and is modulated by the oxygen gradient, membrane permeability, and blood flow in both circulations.

The placenta serves as a critical interface between mother and fetus enabling gas and nutrient exchange. The human placenta is enclosed by the chorionic plate on the fetal side and the basal plate on the maternal side (Fig. 1). Fetal arteries pass through the umbilical cord and branch into the chorionic plate, then enter small structures called villi, which are bathed in maternal blood. Maternal blood perfuses these villi by entering the intervillous spaces via spiral arteries and returning via venous openings that lead back to the uterine veins. Villous veins return the oxygenated blood to the fetus via the umbilical vein. The placenta is divided into cotyledons with varying numbers of villi and a varying vascular supply^5^. Flow in both fetal capillaries and the intervillous space enables exchange of nutrients and waste products between the mother and the fetus^6^. In monochorionic diamniotic twin placentas, each twin is enclosed in its own amniotic sac. The placentas share one chorionic plate, with two umbilical cord insertions, one in each amnionic sac, and one basal plate, thus one maternal blood pool (Fig. 1A). Although fetal vessels of the twins might form anastomoses, in the absence of the twin-to-twin transfusion syndrome^5^, each twin can be considered to have its own placental unit.

Previously, gadolinium based contrast agents have been used to assess placental function in primates, providing identification of maternal perfusion domains and associated regional maternal blood flow^7^. In human singleton placentas associated with severely growth restricted fetuses, dynamic contrast enhancement (DCE) revealed slow, patchy placental perfusion compared to controls^8^. However, given concerns that fetal exposure to gadolinium maybe associated with a higher risk of adverse outcomes^9^, alternate methods to assess placental perfusion are desired. Blood-oxygen-level-dependent (BOLD) MRI with hyperoxia is a promising alternative to gadolinium based approaches as it has been used in prior human studies to measure cerebral perfusion and blood volume^10^.

BOLD signal change in the placenta is affected by maternal blood in the intervillous space (volume fraction 40%) and fetal blood in the fetal capillaries (volume fraction 10%)^11^. Since neither compartment is saturated by oxygen under ambient conditions (80% and 60% respectively)^12^, when oxygenation level increases, deoxygenated hemoglobin [dHb] content drops, causing a BOLD signal increase due to a lower blood R2*. BOLD MRI has been suggested as a potential tool for visualizing placental oxygenation and BOLD MRI signal increases in the placenta and fetal organs due to maternal hyperoxia have been previously reported^13–15^. However, prior BOLD MRI hyperoxia studies of pregnant women focused on the average organ signal change and did not capture important spatiotemporal variations. Moreover, no quantitative biomarkers, such as perfusion associated with placental function have been investigated in previous BOLD studies.

In this paper we perform detailed spatiotemporal analysis of the BOLD MRI contrast generated by transient elevation of blood oxygen saturation, derive an image-based biomarker and test its performance in monochorionic diamniotic twin placentas against important clinical indicators such as fetal brain and liver volume as well as birth weight. The naturally matched genetic growth potential, maternal environment and gestational age of co-twins remove these confounding factors and facilitate interpretations of the findings. The proposed non-invasive method for characterizing maternal placental perfusion on a regional basis with a parameter more directly related to regional placental vascularity and gas exchange represents an important step towards quantifying the relationship between *in vivo* placental perfusion, *in vivo* nutrient transport and fetal growth in humans.

## Results

### Regional placental signal change with maternal hyperoxgenation

**Figure 2:**
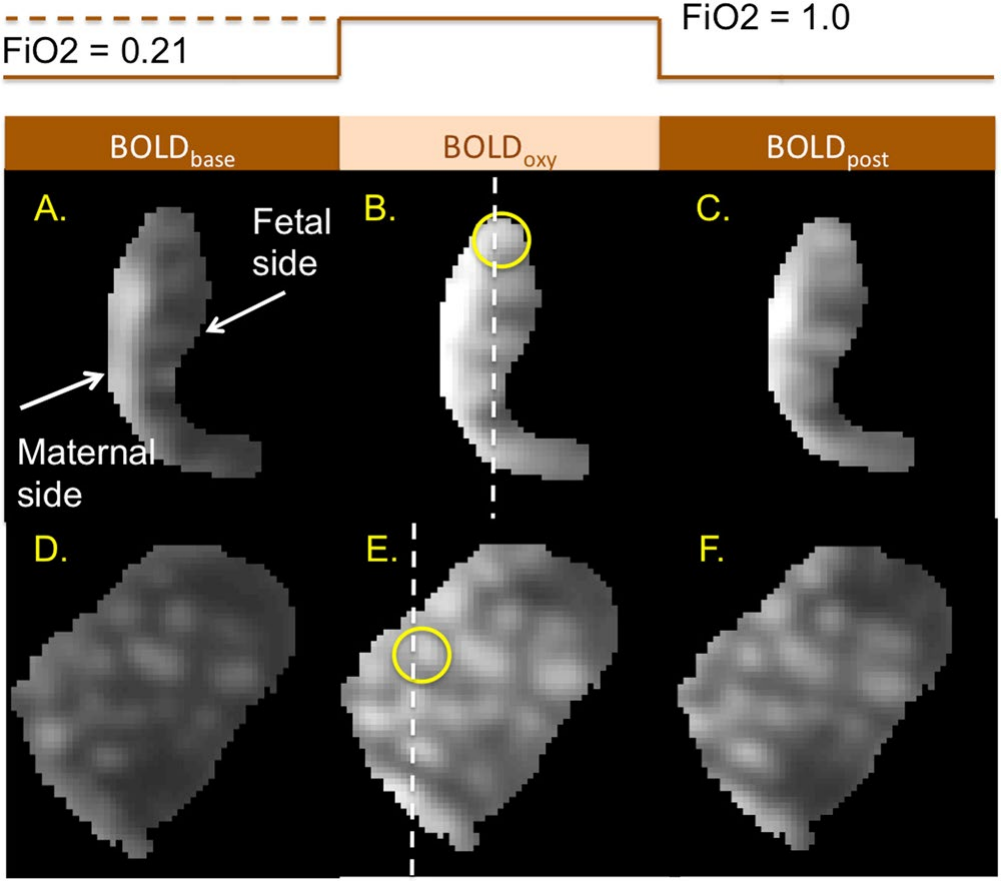
Example cross-sections of a placenta (gestational age 31.4 weeks) during different stages of maternal hyperoxia. (**A**–**C**) BOLD images of the same cross-section view, at baseline (Fraction of inspired oxygen, FiO2 = 0.21), during hyperoxia (FiO2 = 1.0), and post hyperoxia. Dashed line in B defines the plane of section for images (**D**–**F)**. (**D**–**F**) cross-sectional views orthogonal to (**A**–**C**) respectively. Note multiple hyperintense regions in a cotyledon-like distribution. The temporal pattern of signal instensity from baseline to hyperoxia and back is further investigated in the cotyledon circled in B and E in Supplementary Materials.

The first step in the placental image processing pipeline is a non-rigid body motion correction to provide good alignment of the deformable placental structure and improved contrast for our spatiotemporal analysis^16^ (Fig. S1). Figure 2 shows a BOLD MRI (gradient recalled echo planar imaging (GRE-EPI), with T2* = 1/R2* weighting) of one placenta at baseline, hyperoxia, and normoxia respectively. In these images, the maternal side of the placenta is on the left and the fetal side on the right. The observed intensity gradient from left to right is seen most dramatically in Fig. 2A. The cotyledon structures characterized by the consistent bright-dark-bright pattern on the fetal side, is best appreciated in Fig. 2D–F, which are views of the placenta perpendicular to those in Fig. 2A–C respectively. BOLD images show dramatic signal intensity changes with maternal hyperoxia as oxygen moves from the maternal to the fetal side of the placenta. This is better appreciated when viewed dynamically (SI Movie).

### Regional placental oxygen delivery timing and placental Pathology

**Figure 3:**
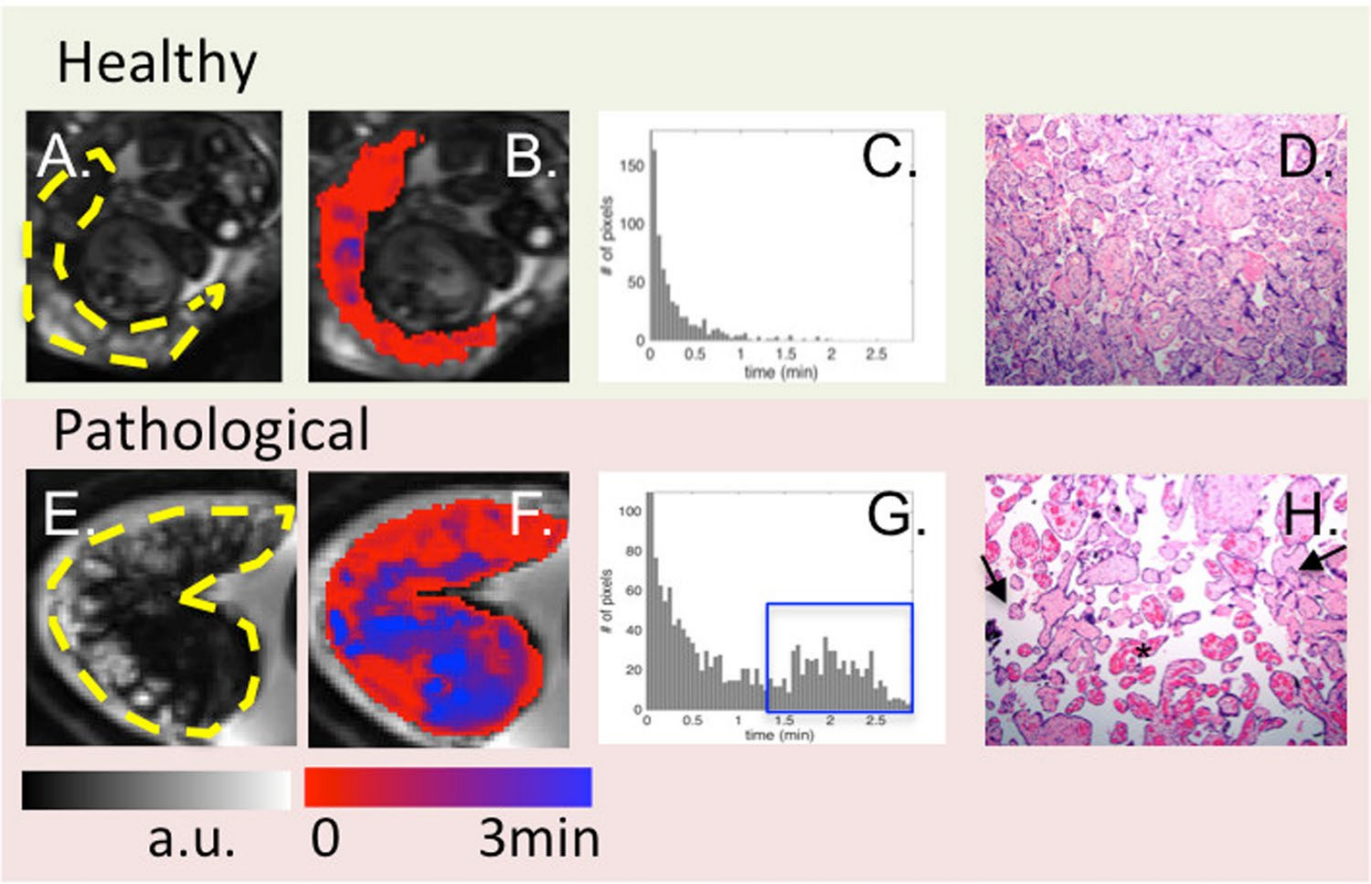
From left to right: BOLD images, TTP maps, histogram of TTP distribution and histology (10X). One control (top) is compared to one case with abnormal placental pathology (bottom). Yellow dashes in A and E outline the placenta. For healthy subjects, TTP values were short and placental histology was normal. For pathological cases, TTP values were longer and less uniform (blue regions in (**F**) and blue box in (**G**)). Arrows in H point to avascular villi and the star identifies chorangiosis.

Regional placental oxygen delivery is modulated by the blood flow in both maternal and fetal circulations, membrane permeability, and oxygen pressure gradient. While there is currently no way to directly quantify oxygen content in the placenta during hyperoxia non-invasively, we utilize the spatiotemporal feature of BOLD MRI time series to quantify the oxygen delivery timing. The dynamic of oxygenation in the placenta was modeled as a step function representing the oxygen exposure paradigm convoluted with a gamma function representing the hemodynamic response. Time-to-plateau (TTP) maps of placentas (i.e. the time that the signal change due to the regional oxygenation reaches to the plateau) were obtained after fitting the signal to the model. For this study, the histopathology was reviewed by an experienced placental pathologist blinded to the MRI findings, as histology is the current gold standard for documenting placental dysfunction. We observed that regional oxygen TTP had a different distribution in placentas confirmed to have moderate to severe placental pathology when compared to healthy placentas. In healthy placentas, the TTP values were near zero and relatively homogeneous across the placenta, with a pattern following that of the cotyledon structure (Fig. 3). In pathological placentas, the TTP values were delayed and inhomogeneous. For example, the placenta shown in Fig. 3 had extensive fetal vascular malperfusion (avascular villi) in the entire placenta as well as chorangiosis in the placental component supplying twin B. The TTP map shows diffusely delayed TTP compared to the TTP distribution in the normal placenta, with the lower lobe (corresponding to twin B) more severely affected. This finding suggests that the vascular proliferation associated with chorioangiosis is inefficient at compensating for poor placental perfusion.

The correlation between the average TTP and our overall pathology severity score was significant across all subjects. Regional TTPs for individual twins with normal or mild placental pathology were significantly shorter than those with moderate to severe pathology (*p* < 0.01). Even after including gestational age as a confounding factor, the association remained significant (*p* = 0.03).

### Regional placental oxygen time-to-plateau compared to fetal brain and liver volumes and birth weight

**Figure 4:**
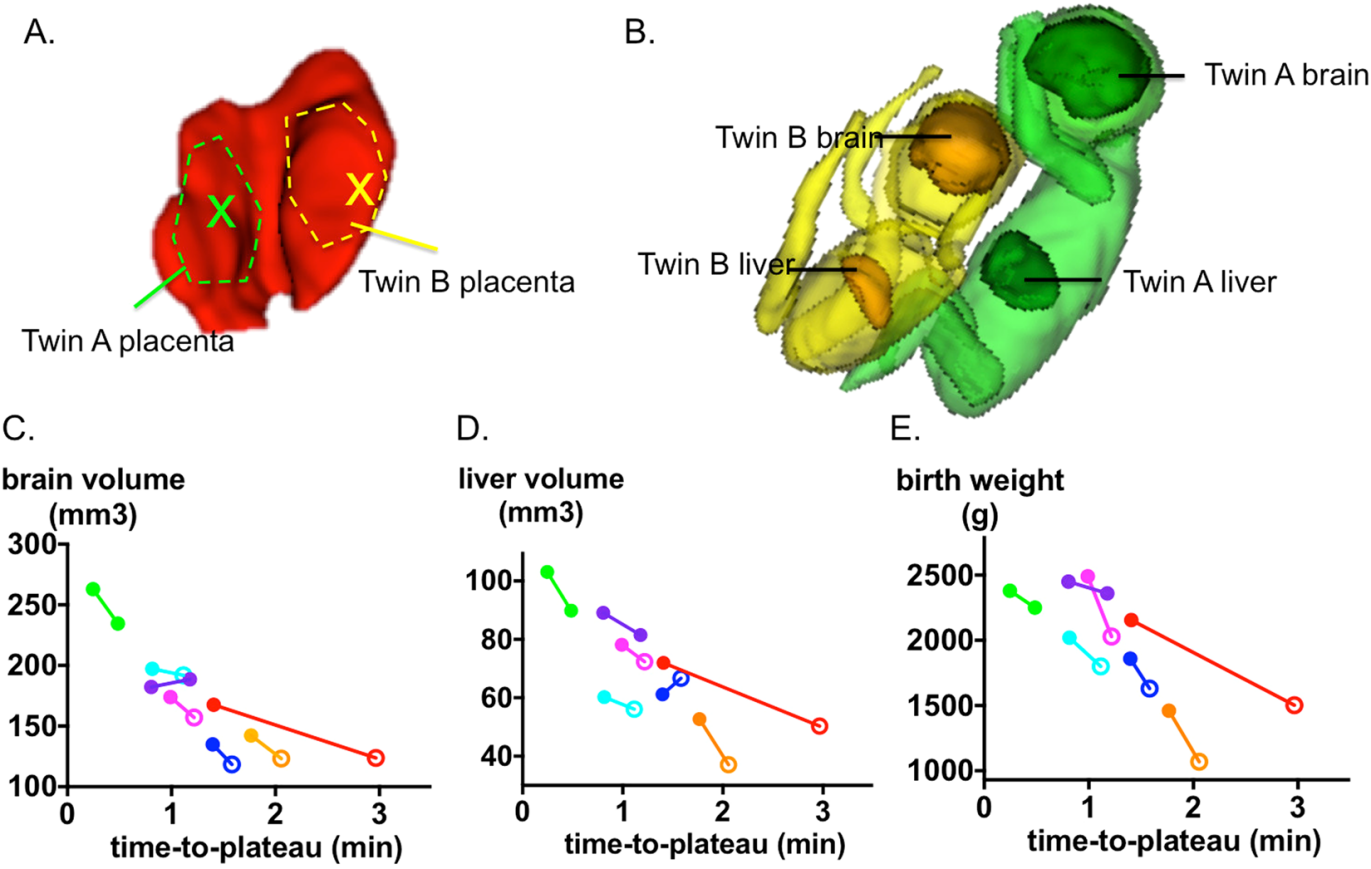
Illustrations of segmentation volumes and of mean time-to-plateau (TTP). (**A**) placenta for the discordant twin pair with indication of ROI segmentation used for the average TTP calculation. (**B**) 3D view of segmented fetal brains and livers in the corresponding discordant twin pair (red points in (**C**–**E**) below). (**C**–**E**) Brain volume, liver volume and birth weight respectively as a function of the average TTP. The brain and liver volume were measured at the time of the scan. Twin pairs are connected by solid line, and are assigned same color. Hollow circles denotes fetuses that proved to be SGA at birth.

We investigated the relationship between placental TTP and measures of fetal growth. The placenta region for each twin was delineated based on umbilical cord insertion, as illustrated in Fig. 4A. The brains and the livers of twin fetuses were segmented on the HASTE images acquired at the time of placental assessment (Fig. 4B) to explore correlations in organ volume and regional placental oxygen delivery. In all twin pairs, covering a spectrum of birth weight discordance from 3.7% to 30.3%, including those born appropriate-for-gestational age (AGA) and those born small-for-gestational age (SGA), the larger placental TTP values were associated with a smaller brain (*p* = 0.06), smaller liver (*p* = 0.06) and smaller body weight (*p* = 0.008), using the Friedman rank sum test. The case illustrated in Fig. 4A,B corresponds to the red points in Fig. 4C–E. In this case, the average TTP for the selected regions was 1.4 min for twin A and 3.0 min for twin B. Twin A had larger brain volume (168 *cm* ^3^ vs. 124 *cm* ^3^) and liver volume (72 *cm* ^3^ vs. 50 *cm* ^3^) compared to twin B. In these twins, the birth weight discordance exceeded 20%^17^, and the placental pathological score was worse (higher) in the smaller twin.

Across subjects, average TTP is significantly correlated with brain volume (*r* = −0.86, *p* = 0.02), and liver volume (*r* = −0.79, *p* = 0.05), using Spearman correlation adjusted for twin pairs (Fig. 4C,D). The correlations remained after applying mixed model regression including gestational age as a co-variate and twin pairs as a random effect (*p* = 0.002 for brain volume vs. TTP and *p* = 0.001 for liver volume vs. TTP). Twin birth weights were recorded at delivery, with time from the fetal MRI scan to birth ranging from 0.2 to 5.5 weeks. Significant correlation (Spearman, adjusted for twin pairs) was found between the average TTP and birth weight (*r* = −0.75, *p* = 0.07) (Fig. 4E). The significance of this correlation improved when mixed model regression was applied including gestational age covariates (gestational age at date of MR scan and time from scan until birth) and twin pairs as a random effect (*p* = 0.0003).

### Comparisons to ultrasound measurements

Routine clinical screening for placental insufficiency focuses on estimated fetal weight (EFW) from ultrasound and Doppler velocimetry of the umbilical arteries (UmA). In this study, when we include both EFW and TTP in the mixed model to predict birth weight, while keeping gestational age as a covariate and twin pairs as a random effect, the TTP showed significance (*p* = 0.02) whereas EFW did not (*p* = 0.9). Similarly when we include both UmA Doppler grade and TTP in the mixed model to predict placenta pathological score, both Doppler and TTP were significant covariates (*p* = 0.01 vs. *p* = 0.03). Therefore TTP predicted birth weight when EFW did not and TTP significantly contributed to determination of pathological score when combined with UmA.

### Fetal response to maternal hyperoxia

**Figure 5:**
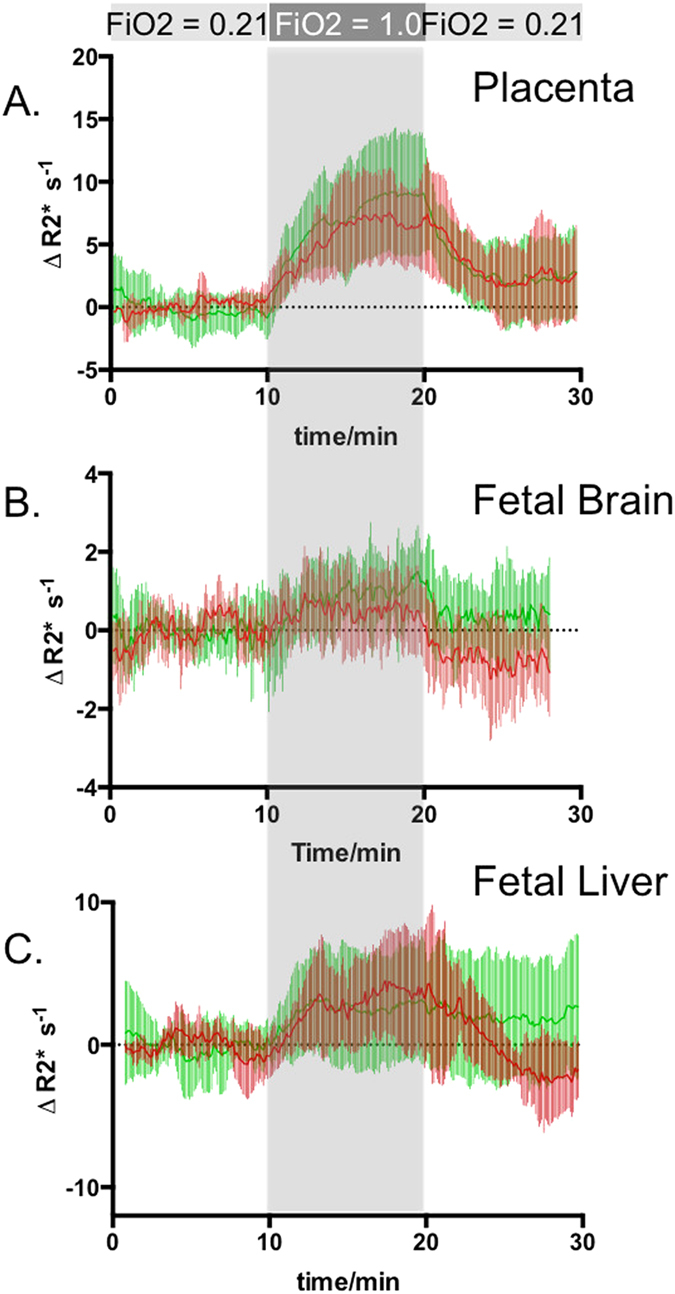
Time activity curves of placentas and fetal organs presented as signal intensity change relative to baseline (ΔR2*). Green: mean and standard deviation of ΔR2* for the organs of AGA fetuses; Red: mean and standard deviation of ΔR2* for the organs of SGA fetuses as defined retrospectively using birth weight. (**A**) placentas, (**B**) fetal livers, (**C**) fetal brain.

The placentas that are associated with SGA fetuses exhibit slower rates of ΔR2* rise from baseline (lower slope) at the beginning of hyperoxia compared to those who prove to be AGA at delivery (ΔR2* per minute: 0.88 *s* ^−1^ vs. 1.89 *s* ^−1^, *p* = 0.04, measured at 10 min) as shown in Fig. 5A. This is consistent with the finding that average TTP was longer in placentas associated with SGA neonates (*p* = 0.03). However, there was no significant difference in the magnitude of signal change (ΔR2*) between the two groups throughout the oxygen paradigm, suggesting that absolute signal differences are insensitive measures.

In corresponding fetal livers, there were no differences in rates of ΔR2* rise at the onset of hyperoxia but significant differences in rate of ΔR2* decrease after termination of hyperoxia were observed (Fig. 5B). After hyperoxia, ΔR2* fell significantly faster in SGA livers (ΔR2* per minute: −0.91 *s* ^−1^ for SGA vs. −0.16 *s* ^−1^ for AGA, *p* = 0.002, measured at 23 min), resulting in a significant difference in R2* at 28 min relative to the baseline (*p* = 0.048). Fetal brains also exhibit a similar behavior, although neither the rates of ΔR2* rise nor fall was significant. However, the ΔR2* became significantly lower in SGA brains by approximately 27 min. Placentas associated with SGA fetuses tended to reach maximum oxygen level before AGA placentas, with the same tendency observed for fetal livers and brains (although these differences were not significant). Interestingly, SGA fetal livers and brains had significantly lower R2* values at the end of the post-hyperoxia phase than at baseline.

## Discussion

This study demonstrates a novel application of BOLD MRI for assessment of placental insufficiency. We investigated the utility of time-to-plateau (TTP) of the BOLD MRI time series under maternal hyperoxia as a surrogate marker for placental oxygen transport. We demonstrated that 1) placental TTP maps depict a regional perfusion pattern consistent with the known cotyledon structure; 2) placental TTP was significantly longer in placentas with moderate to severe pathology compared to normal to mild pathology; 3) placental TTP within each twin pair has a consistent tendency to be longer in the smaller twin; 4) placental TTP is significantly correlated with liver and brain volume and birth weight across all subjects, with TTP of fetuses destined to be SGA at birth significantly longer than those destined to be AGA. These findings suggest that TTP with maternal hyperoxia is a promising *in vivo* biomarker of placental function.

Regional and temporal variations in TTP (Fig. 3) agree with known regional placental anatomy. For example, TTP exhibits a significant negative correlation with gestational age (p = 0.002) that suggests an increase in placental perfusion with increasing gestational age. This is consistent with the increased dilation of the spiral arteries as gestation transitions from first to the second trimester^18^. As a result, uterine blood flow^19^ continues to increase linearly from 20 to 38 weeks, with an associated exponential increase in umbilical venous blood flow from 20 weeks to term^20, 21^. Given the small range of gestational ages in this study (29–34 wks), linear mixed model is applied when accounting for gestational age as a co-variate. A more detailed exploration of placental TTP across gestation in healthy twin and singleton pregnancies is needed to fully characterize TTP dependence on gestational age.

In pathological placentas, TTP maps clearly show localized regions with severely delayed TTP and a larger range of TTP values compared to normal (Fig. 3). These findings are consistent with a prior gadolinium based study of growth restricted singletons^8^. In addition, placental average TTP values obtained *in vivo* were in agreement with postnatal pathological assessment, the current gold standard for documenting placental dysfunction. Although there was a time lapse between imaging and placental examination (Table S1), we cannot exclude extension of the pathologies over time. The findings reported herein are those of chronic insult and likely occurred long before the delivery. We did not find any acute pathologies, e.g., acute villous edema, meconium pigment, or acute chorioamnionitis. Such spatiotemporal information of placental pathophysiology is impossible to obtain with current ultrasound systems. Thus TTP provides regional information that may allow physicians to monitor placental function longitudinally with more sensitivity. More detailed correlation of regional placental TTP and histopathology is required to fully characterize the sensitivity and specificity of TTP for different types of placental pathology.

In monozygotic twins, the twin pairs are most optimally matched for genetic growth potential, gestational age and maternal environment. As a result, discordant twin growth can be attributed primarily to differences in regional uterine and placental function. This is an example of environmental influences impacting fetal growth. Using the average TTP of each placenta as an indicator of placental oxygen transport, the smaller of the discordant twins (>20% different in estimated weight by fetal US) was associated with a significantly longer average TTP. In addition, all seven smaller twins were associated with a longer placental average TTP. In addition we observed a significant association between TTP and gestational age. Thus TTP appears to detect a spectrum of placental function that correlates with fetal size as well as gestational age. Therefore, TTP is a promising new technique for assessing placental health as a function of gestational age.

Quantitative analysis of oxygen concentration changes is challenging because BOLD signal is dependent on many biophysical and physiological parameters. The magnitude of placental ΔR2* during hyperoxia reported previously in studies performed at 1.5 T differs between studies, for example, ΔR2* = 4.30 ± 1.82 *s* ^−1^ in one study^22^ and 2.83 ± 0.63 *s* ^−1^ in another study of healthy controls^13^ (calculated based on reported % signal change). In our study ΔR2* = 9.83 ± 4.49 *s* ^−1^ measured during the last 2 min of hyperoxia for healthy subjects, is much larger than literature values primarily because we image at the higher field strength of 3 T instead of 1.5 T. The increased field strength increases R2* relaxivity and therefore when oxygen binds with deoxyHb, there is a more dramatic decrease in R2*. Regional placental physiology can also influence ΔR2* with hyperoxia. For example, in our study pathological placentas had smaller ΔR2* change during hyperoxia (6.55 ± 3.30 *s* ^−1^) compared to healthy placentas (although not significant). This is in agreement with observations in intrauterine growth restricted mice models vs. control^23, 24^ and a few cases reported in a human study^15^. In addition, multiple physiological factors such as hematocrit levels of the blood, binding affinity of fetal hemoglobin and baseline oxygenation levels could affect the capacity for ΔR2* change during hyperoxia. MR oximetry based on T2 mapping could quantify oxygenation level in blood vessels^12^, however regional placental data are lacking. Quantitative measurements of R2* show promise in normal primate studies^25^ and may improve our ability to detect consistent changes in ΔR2* with placental pathology. However, due to the challenges of quantitative R2* imaging in humans we focused on TTP.

Oxygen delivered from the placenta to the fetus diffuses freely into fetal organs, therefore monitoring fetal organ ΔR2* with maternal hyperoxia provides information on oxygen transport across placenta to the fetus. To understand the observed differences in ΔR2* of the placenta and fetal organs (Fig. 5), differences in regional blood volume fraction and potential blood volume changes during hyperoxia must be considered. The markedly different magnitudes of ΔR2* observed in the placenta, fetal liver and fetal brain are likely due to the markedly different blood volume fraction of these organs. As expected, the brain shows the smallest |ΔR2*|_*max*_ change, (1.65 ± 0.97 *s* ^−1^) as it has a much smaller blood volume fraction. The ratio of |ΔR2*|_*max*_ for placenta, fetal liver and fetal brain are roughly 6:3:1 respectively, which is consistent with the relative blood volume fraction of the placenta being much larger than the fetal liver and even larger still than the brain.

Changes in human fetal brain R2* with maternal hyperoxia have not been previously reported. While in fetal sheep and mice brain R2* signal changes have been observed during maternal hyperoxia at 1.5 T and 4.7 T^23, 24^, R2* signal change was not observed in human fetal brains at 1.5 T^14, 26^. It is unclear if the magnitude of R2* change is minimized by vasoconstriction during hyperoxia. Human fetal Doppler studies of middle cerebral arteries during maternal hyperoxia report measureable but inconsistent changes that support both vasodiliatation and vasoconstriction^27–29^. Here, significant increases, albeit small, were detected in fetal brains likely due to the increased field strength (3 T) and more sophisticated motion mitigation strategies. How these signal changes are modified by changes in fetal cerebral vasoreactivity with gestational age remains unclear.

The decreases in R2* below baseline in the livers and brains of fetuses destined to be SGA, but not their corresponding placenta, is intriguing. This is particularly interesting, given that the placenta consumes a significant amount of oxygen with normal term placenta, more than 40% of the overall oxygen delivered to uterus^30, 31^. One possibility is that in pathological placentas, the rate of oxygen consumption may not remain constant. Similar to high altitude pregnancies, where there is chronic low ambient pO2, the placental metabolism may adjust to reduce its own oxygen consumption by switching from oxidative metabolism of carbohydrates to anaerobic glycolysis^32^. In this chronic case, the fetus is provided with lower O2 and glucose supply, slowing fetal growth^32–34^. We hypothesize a similar physiology occurs in this study, with the insufficient placenta maintaining a lower baseline oxygen consumption due to chronic hypoxia. With maternal hyperoxia, placental oxygen extraction may increase and continue at a higher rate before the oxygen returns to baseline, resulting in the observed R2* drop in fetal organs below baseline. The lack of undershoot in the organs of fetuses destined to be AGA is consistent with the observation that in healthy pregnancies, oxidative metabolism in the placental-fetal unit remains constant during acute fluctuations of oxygenation^35^. If our hypothesis is true, a transient hyperoxia episode may prove useful in probing the baseline metabolic state of the placenta. This hypothesis, although preliminary, deserves further study. Recent advances in ^13^C imaging of placenta^36^ may provide additional complimentary information on placental metabolic shifts with oxygenation.

Accurate determination of placental volume for each twin was not possible, as identification and validation of the equator location is not feasible in this study. The patient population is small, as discordant monochorionic twins are not common, and recruitment was challenging. Nonetheless, we believe the information gained by having fetuses matched for genetics, gestational age and maternal environment enabled us to isolate the effects of placental dysfunction.

In summary, in our BOLD imaging study with maternal hyperoxia, average placental TTP correlated positively with placental histopathological score at birth, negatively with fetal brain and liver volume at time of MRI and negatively with birth weight. In monozygotic twin pairs, where the genetic growth potential, gestational age and maternal environment are optimally controlled, we show that placental oxygen TTP is consistently longer in the smaller of the discordant twin pairs. These results expand existing knowledge of BOLD MRI with maternal hyperoxia to demonstrate not only global but also regional placental signal changes with introduction of TTP as a quantitative parameter. In this study group, we also show that TTP is better than US biometry and umbilical artery Doppler grade at predicting birth weight, and when combined with umbilical artery Doppler grade improves prediction of placental pathology scores. Thus BOLD MRI measures of TTP with maternal hyperoxia is a promising *in vivo* measure of regional placental function.

## Materials and Methods

### Statement

All experimental protocols were approved by a named institutional/licencing committee. Specifically, MRI scan on pregnant mothers, ultrasound data collection from obstetric clinical record, follow up birth record collection and pathological reports collection were approved by the Instutional Review Board (IRB) at the Boston Children’s Hospital. Informed consent was obtained from all subjects, and all methods were carried out in accordance with the relevant guidelines and regulations of IRB, Protocol #00012586.

### Subjects

This study enrolled seven pairs of monochorionic diamniotic twins, whose gestational age ranged from 29 to 34 weeks. Exclusion criteria: Twin-to-Twin Transfusion Syndrome, fetal anomalies, maternal hypertension and any diabetes. Of the 7 study subjects, 6 were Caucasian and one was Asian. The median age was 34 with a range of 28 to 42 and the median height was 163 cm with a range of 157 to 170 cm. The median BMI was 23.3 with a range of 21.2 to 27.6, with only two of the patients being overweight and none being obese. Table S1 reports demographics of the population, prenatal and postnatal data.

### Image acquisition

MRIs were acquired on a 3 T Skyra scanner (Siemens Healthcare, Erlangen, Germany) using a combined 18-channel body and 12-channel spine-receive arrays, while mother remained in left lateral position. HASTE images (2 × 2 × 5 *mm* ^3^) of the whole uterus was acquired for anatomical reference. BOLD imaging of the whole uterus was collected using single-shot gradient echo EPI sequence with field-of-view (FOV) and matrix size adjusted to achieve in plane resolution of 3 × 3 *mm* ^2^, slice thickness of 3 mm, interleaved; *TR* = 5–8 *sec*, *TE* = 32–38 *ms*, *FA* = 90 deg, *BW* = 2.3 *kHz*/*px*. The number of time frames was adjusted so that the total acquisition time was 30 min. SAR did not exceed 2 W/kg at any time. Oxygen supply to the mother was alternated from room air with fraction of inspired oxygen (FiO2) of 21%, to oxygen (flow rate 15 L/min, FiO2 100%), and back to room air via non-rebreathing facial mask during MRI acquisition. Each epoch was 10 min long.

### Image processing

The acquired images were first corrected for signal non-uniformity and then registered to mitigate motion with a non-rigid transformation approach designed for BOLD time series which outperforms traditional rigid body motion correction approaches^16^ (Fig. S1). The bias field accounting for B1 inhomogeneities was estimated from averaged selected volumes collected in first 10 minutes by using N4ITK algorithm^37^, and subsequently applied to correct for signal non-uniformity of each volume. For intravolume motion correction, each volume was separated into two sub-volumes: even and odd slices, then registered to each other using the group-wise approach^38^. For intervolume motion correction, a reference volume with a least sum of mean square error (MSE) difference compared to the rest of the volumes in the series was selected. Then pairwise registration was employed between the reference volume and the other volumes. As an initialization, a six degrees of freedom rigid transformation as a mapping from the reference volume to the rest of the volumes was estimated. To compensate for motion of the deformable fetal body and placenta in the time series, a non-rigid body transformation was performed following the initial rigid body alignment. To compensate for motion of the brain, which is more rigid, a second rigid body transformation was estimated following the initial rigid body alignment. All registration steps were carried out in Elastix^39^, an open source software. ROIs of the placenta and fetal organs in the reference frame were manually delineated using ITK-SNAP^40^, and propagated to all time frames. Outlier volume detection was performed based on 1) over-deformation during intravolume motion correction; and 2) unexpected average signal intensity change of nearby time frames. The median fraction of outliers discarded for placenta was 3.3% (0.9–53.4%), for fetal brain was 22.1% (1.6–85%) and for fetal liver was 15.6% (1.4–34.8%). The registered 4D placenta image was subsequently smoothed in the spatial domain by a Gaussian kernel with width of 5 pixels, sigma 1.5, and interpolated in the temporal domain followed by a smoothing window of around 1 min (8–12 TRs). The indicator of oxygen saturation level change, ΔR2*, was calculated for placenta and fetal organs, as follows:

1 $$\begin{array}{rcl}{\rm{\Delta }}R{2}^{\ast }(t) & = & -(R{2}^{\ast }-R{{2}^{\ast }}_{baseline})\\  & = & \,\mathrm{log}(S(t)/{S}_{baseline})/TE\\  & = & -r{2}^{\ast }\cdot ({V}_{F}\cdot {\rm{\Delta }}[dH{b}_{F}(t)]+{V}_{M}\cdot {\rm{\Delta }}[dH{b}_{M}(t)])\end{array}$$

where r2* is the relaxivity of deoxygenated hemoglobin, V_F_ and V_M_ are the volume fractions of fetal and maternal blood, [dHb_M_], [dHb_F_] stands for the maternal and fetal deoxygenated hemoglobin concentration repectively. When oxygenation level increases, [dHb] drops, which decreases R2* and increases BOLD signal S(t), therefore we define ΔR2* in the way that it increases with the oxygenation level increase.

Using ITK-SNAP, placenta regions corresponding to each twin were determined by tracing umbilical cord that connects each fetus to its placenta. The placental tissue corresponding to each twin was manually delineated by first identifying the cord insertion for each twin and confirming with an expert radiologist. Second, as the boundary dividing portions of the placenta supplying each twin is not visible, a 5 cm boundary zone half-way between the two cords was excluded from analysis. ROIs from each cord to the respective boundary were extended circumferentially around each cord to define placental regions corresponding to each twin. The fetal brain and liver was manually delineated with boundaries supervised and confirmed by expert radiologists.

Similarly to the fMRI studies in the brain^41, 42^, we employ the Gamma function to capture the shape of the hemodynamic response of the placenta. Gamma function fitting was implemented both in the regions-of-interest (ROIs) and on voxel-by-voxel basis. The R2* time series in placental tissue are fit to a gamma function convolved with the oxygen paradigm using Least-Squares:

2 $$\begin{array}{rcl}R\mathrm{2\ast (}t|\alpha ,\beta ,{\rm{\Delta }},C\mathrm{1,}C\mathrm{2)} & = & C1+C2\cdot {(t-{\rm{\Delta }})}^{\alpha -1}\cdot {e}^{-(t-{\rm{\Delta }})/\beta }\otimes {P}_{oxy}(t,{\rm{\Delta }})\\ {P}_{oxy}(t,{\rm{\Delta }}) & = & \{\begin{array}{lll}1 & {\rm{for}} & t\ge {\rm{\Delta }}\\ 0 & {\rm{for}} & t < {\rm{\Delta }}\end{array}\}\end{array}$$

3 $$\tau =\alpha \cdot (\beta -1)$$

4 $$TTP={\rm{\Delta }}+\tau $$

where x is the frame number after onset of signal change of the R2* time series, *α* and *β* are gamma function parameters, C1 describes baseline T2* weighted signal, and C2 describes the amplitude of R2* change; P_*oxy*_ is a step function that describes administration of oxygen. With Δ we describe the delay time of oxygen arrival; together with the mode *τ* of the Gamma function (derived from *α* and *β*), we obtain Time-to-Plateau (TTP). Variations in fitting and TTPs are demonstrated in Table. S3.

### Outcome Metrics

Ultrasound antenatal measurements included estimated fetal weight and Doppler velocimetry of the umbilical artery. MRI measurements included fetal brain and fetal liver volumes were measured based on delineation of HASTE images. Birth record: Apgar scores at 0 and 5 minutes were collected. All babies’ Apgar scores were in the normal range (7–10), indicating normal respiratory and cardiac function. Birth weights were collected for all subjects. Appropriate for gestational age (AGA) was defined as larger than 10th percentile, and SGA was defined as equal to or smaller than 10th percentile. Birth weight discordance was defined as the fraction of difference between co-twin birth weights divided by the weight of the larger twin.

Placental gross inspection was performed as part of clinical care by experienced pathologists and technicians without knowledge of the MRI findings. The placenta lobes (A or B) corresponding to each twin fetus were noted according to clamps on the attached umbilical cords. Tissue specimens were then sampled and send for routine hematoxylin and eosin stained slides. For this study, the histology was reviewed by a single experienced placental pathologist also blinded to the MRI findings. Global histopathological scores were assigned based on a 4-point scale from 1 = normal placental histology to 4 = severely abnormal placental histology. The numerical score was based on both the extent and severity of the pathology. Minor findings included chorangiosis in a normal weight placenta, placental weights <10th or >90th percentiles for gestational age, or low grade fetal vascular malperfusion. 1 minor finding received a score of 1. Two minor findings received a score of 2. Moderate pathological findings included high grade fetal vascular malperfusion, diffuse chorangiosis, fetal normoblastemia, extensive increase in perivillous fibrin, or placental infarct(s) of more than 5% of the placental mass. Moderate pathologic findings received a score of 2. Three minor or one minor and one moderate finding received a score of 3. Severe pathologic findings included 3 minor or two moderate findings and received a score of 4. There were no cases of inflammatory placental pathology (e.g. chorioamnionitis or villitis)^43^.

### Statistical Analysis

Statistical analyses were performed in Matlab^®^9.1, (MathWorks Inc., Natick, MA 2016). The Friedman ranksum test was used for testing the relationship between the difference in fetal growth and their corresponding placental TTP within each twin pair. For each twin pair, for each of the two quantities to be correlated, one of the two values was selected at random, and a Spearman correlation was calculated. This was repeated 1000 times, and the median correlation and its associated p-value was reported. Mixed models were used for across subjects regression analyses, with mother’s ID included as a random effect to account for correlation between fetuses from the same mother. Because of long-range temporal correlations, we modeled the temporal data using functional data analysis methods as implemented in the FDA toolbox in Matlab^44^. We used a cubic B-spline basis with knots at two-minute intervals, then calculated mean and standard deviation as functions of time for AGA and SGA groups, and t-statistic and p-value functions comparing the group means.

## Electronic supplementary material

Supplementary Materials

Movie S1
