# Supplementary material for: In Vivo Quantification of Placental Insufficiency by BOLD MRI: A Human Study
Source: Sci Rep. 2017 Jun 16;7:3713. doi: 10.1038/s41598-017-03450-0 (PMC5473907; doi:10.1038/s41598-017-03450-0)
Supplement: Supplementary file 1 — Supplementary Materials [file 41598_2017_3450_MOESM1_ESM.doc]

Title: *In Vivo* Quantification of Placental Insufficiency by BOLD MRI: A Human Study

**Authors:** Jie Luo^1,2,†^, Esra Abaci Turk^1,2,†^, Carolina Bibbo^3^, Borjan Gagoski^1^, Drucilla J. Roberts^4^, Mark Vangel^5^, Clare M. Tempany-Afdhal^6^, Carol E. Barnewolt^7^, Judy A. Estroff^7^, Arvind Palanisamy^8^, William H. Barth^9^, Chloe Zera^3^, Norberto Malpica^2,10^, Polina Golland^11,12^, Elfar Adalsteinsson^2,11,13^, Julian N. Robinson^3,‡^, Patricia E. Grant^1,‡,*^

^*^To whom correspondence should be addressed. E-mail: [ellen.grant@childrens.harvard.edu](mailto:ellen.grant@childrens.harvard.edu)

^†^J.L and E.A.T. contributed equally to this work.

^‡^J.N.R and P.E.G. contributed equally to this work.

**Affiliations:**

^1^Fetal-Neonatal Neuroimaging and Developmental Science Center, Boston Children’s Hospital.

^2^Madrid-MIT M+Vision Consortium, RLE, Massachusetts Institute of Technology.

^3^Maternal Fetal Medicine, Brigham and Women’s Hospital.

^4^Pathology, Massachusetts General Hospital.

^5^Radiology, Massachusetts General Hospital.

^6^Radiology, Brigham and Women’s Hospital.

^7^Radiology, Boston Children’s Hospital.

^8^Anaesthesia, Brigham and Women’s Hospital.

^9^Obstetrics and Gynecology, Massachusetts General Hospital.

^10^Medical Image Analysis and Biometry Laboratory, Universidad Rey Juan Carlos.

^11^Electrical Engineering and Computer Science, Massachusetts Institute of Technology.

^12^Computer Science and Artificial Intelligence Laboratory, Massachusetts Institute of Technology.

^13^Institute for Medical Engineering and Science, Massachusetts Institute of Technology.

**Supplementary Materials**


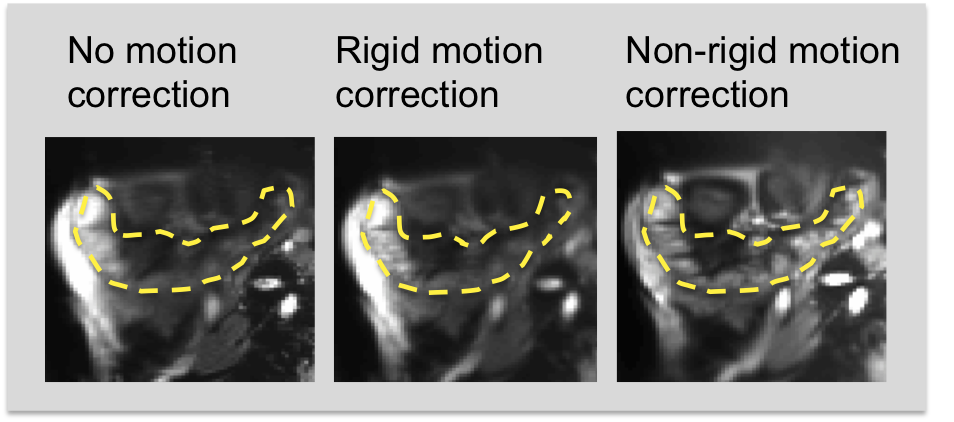


Fig. S1. Image of placenta from averaged time series. Yellow dashed line outlines the placenta region. Anatomical structure is visually more crisp by application of non-rigid motion correction approach (right), compared to the data without motion correction (left) or to the one with rigid body motion correction (middle).

Fig. S2. Illustration of TTP distribution in the placenta. a) Histogram of TTP values of two placenta volumes corresponding to twins of subject #1, blue values represent histogram of larger twin and magenta the smaller twin. Note the much longer TTP values in the placenta of the smaller twin d) Histograms of TTP values of two placenta volumes corresponding to twins of subject #4, blue values represent histogram of larger twin and magenta the smaller twin. Note that these twins were similar in size and the histograms are more similar than those of subject #1.

Table S1. Summary of prenatal ultrasound data, MRI metrics and postnatal data for all subjects.

|  |  | **Ultrasound** | | | **MRI** | | | | **Birth Records** | |  |
| --- | --- | --- | --- | --- | --- | --- | --- | --- | --- | --- | --- |
| ID | Maternal factors* | EFW (percentile) | Doppler** | GA (US) | Brain (mm3) | Liver (mm3) | TTP (std.dev.) | GA (MR) | Birth weight (percentile) | GA (BW) | Path score*** |
| 1A | 35 / C/ | 1335 (01) | Normal | 31.6 | 167.6 | 72.0 | 1.41 (0.57) | 31.4 | 2155 (23) | 35.7 | 3 |
| 1B | 157/57.2 | 998 (00) | AED | 31.6 | 123.7 | 50.3 | 2.97 (0.62) | 31.4 | 1503 (01) | 35.7 | 4 |
| 2A | 34 / A/ | 1187 (40) | Normal | 28.1 | 142.2 | 52.7 | 1.76 (0.34) | 29.0 | 1460 (48) | 31.0 | 1 |
| 2B | 157/57.8 | 925 (02) | AED | 28.1 | 123.2 | 37.1 | 2.06 (0.38) | 29.0 | 1070 (06) | 31.0 | 4 |
| 3A | 38 / C/ | 1499 (23) | Normal | 30.6 | 193.0 | 81.5 | 1.18 (0.73) | 31.4 | 2359 (32) | 36.1 | 2 |
| 3B | 165/59.4 | 1683 (54) | Normal | 30.6 | 181.3 | 89.1 | 0.80 (0.67) | 31.4 | 2450 (38) | 36.1 | 1 |
| 4A | 33 / C/ | 1701 (01) | Normal | 33.8 | 234.5 | 89.8 | 0.49 (0.67) | 34.6 | 2250 (39) | 34.7 | 1 |
| 4B | 163/62.4 | 2197 (33) | Normal | 33.8 | 263.0 | 103.0 | 0.25 (0.59) | 34.6 | 2380 (53) | 34.7 | 1 |
| 5A | 28 /C | 1960 (04) | Normal | 34.6 | 197.2 | 60.3 | 0.82 (0.61) | 34.6 | 2020 (11) | 35.6 | 1 |
| 5B | 157/66.2 | 1774 (01) | AED | 34.6 | 192.0 | 56.1 | 1.12 (0.62) | 34.6 | 1800 (04) | 35.6 | 3 |
| 6A | 33 / C | 1624 (31) | Normal | 31.0 | 134.9 | 60.2 | 1.40 (0.54) | 31.0 | 1860 (25) | 34.0 | 2 |
| 6B | 170/62.4 | 1293 (01) | Normal | 31.0 | 118.4 | 66.7 | 1.58 (0.60) | 31.0 | 1630 (09) | 34.0 | 2 |
| 7A | 42 / C | 1459 (06) | Normal | 31.3 | 157.1 | 72.4 | 1.22 (0.59) | 31.3 | 2030 (07) | 36.9 | 2 |
| 7B | 166/75.8 | 1582 (18) | Normal | 31.3 | 173.9 | 78.2 | 0.99 (0.62) | 31.3 | 2490 (29) | 36.9 | 2 |

EFW = estimated fetal weight in gram * Maternal age (years) / ethnicity (C for Caucasian, A for Asian)/ height (cm) / weight (kg) ** AED = absence of end-of-diastolic flow; TTP = time-to-plateau; *** normal = 1, mild = 2, moderate = 3, severe = 4; GA = gestational age

Table S2. Number of Outliers for each subject

| Subject Number | Placenta | Brain A | Brain B | Liver A | Liver B |
| --- | --- | --- | --- | --- | --- |
| 1 | 53.39% | 34.75% | 85.17%* | 31.78% | 34.75% |
| 2 | 3.27% | 11.84% | 1.63% | 24.49% | 9.80% |
| 3 | 10.69% | 29.31% | 30.69% | 19.66% | 14.14% |
| 4 | 2.35% | 19.94% | 24.34% | 4.99% | 3.81% |
| 5 | 1.69% | 14.24% | 10.85% | 1.36% | 9.15% |
| 6 | 0.89% | 6.70% | 15.18% | 6.25% | 15.18% |
| 7 | 12.77% | 38.72% | 32.77% | 25.11% | 17.45% |

* We could not use the whole time series as a result of the outlier detection.

Table S3. Variations in TTP fitting and TTP values.

| ID | Mean Correlation Coefficient (std.dev.) | TTP  (std.dev.) |
| --- | --- | --- |
| 1A | 0.90 (0.11) | 1.41 (0.57) |
| 1B | 0.82 (0.17) | 2.97 (0.62) |
| 2A | 0.80 (0.19) | 1.76 (0.34) |
| 2B | 0.84 (0.18) | 2.06 (0.38) |
| 3A | 0.87 (0.09) | 1.18 (0.73) |
| 3B | 0.84 (0.11) | 0.80 (0.67) |
| 4A | 0.93 (0.04) | 0.49 (0.67) |
| 4B | 0.92 (0.06) | 0.25 (0.59) |
| 5A | 0.90 (0.13) | 0.82 (0.61) |
| 5B | 0.88 (0.10) | 1.12 (0.62) |
| 6A | 0.76 (0.16) | 1.40 (0.54) |
| 6B | 0.77 (0.14) | 1.58 (0.60) |
| 7A | 0.88 (0.09) | 1.22 (0.59) |
| 7B | 0.93 (0.08) | 0.99 (0.62) |

Movie S1.

BOLD signal level increase in placenta in real time during oxygen paradigm. Movie on the left displays cross section of a healthy placenta shown in Fig. 2 (rest of the image masked out in order to highlight the placenta) after registration. Plot on the right shows normalized average BOLD signal of the whole placenta as a function of time. As the movie plays, the cursor on the plot is synchronized with the frames on the left.
